# Prognostic value of HHLA2 expression in solid tumors: A meta-analysis based on the Chinese population

**DOI:** 10.1097/MD.0000000000026789

**Published:** 2021-07-30

**Authors:** Chuanmeng Zhang, Jie Xu, Jun Ye, Xiaohong Zhang

**Affiliations:** a The Center for Translational Medicine, Taizhou People's Hospital, Affiliated 5 to Nantong University, Taizhou, Jiangsu Province, China.; b Outpatient Department, Taizhou People's Hospital, Affiliated 5 to Nantong University, Taizhou, Jiangsu Province, China.

**Keywords:** HHLA2, meta-analysis, overall survival, solid tumor

## Abstract

**Background:**

Human endogenous retrovirus-H long terminal repeat-associating protein 2 (HHLA2), a newly discovered member of the B7 family, is overexpressed in numerous tumors. However, the prognostic impact of HHLA2 in human cancers remains controversial. Thus, we performed this meta-analysis to explore the prognostic value of HHLA2 in Chinese patients with solid tumors.

**Methods:**

PubMed, Embase, Web of Science, Chinese National Knowledge Infrastructure, and WanFang databases were systematically searched for eligible studies that evaluated the impact of HHLA2 on overall survival (OS) in patients with cancer. Hazard ratios (HRs) and 95% confidence intervals (CIs) were combined to evaluate the association between HHLA2 expression and OS in solid tumors. Odds ratios (ORs) and 95% CIs were pooled to assess the correlation between HHLA2 expression and clinicopathological characteristics in solid tumors.

**Results:**

A total of 12 studies, including 15 cohorts and 1747 patients, were included in this meta-analysis. We found that high HHLA2 expression was significantly associated with shorter OS (HR = 1.65, 95% CI: 1.12–2.43). Subgroup analysis by cancer type demonstrated that high HHLA2 expression was associated with poor OS in patients with clear cell renal cell carcinoma (HR = 3.42, 95% CI: 2.39–4.91), gastric cancer (HR = 2.03, 95% CI: 1.31–3.16), intrahepatic cholangiocarcinoma (HR = 1.77, 95% CI: 1.24–2.53), lung cancer (HR = 2.14, 95% CI: 1.33–3.44) and other cancer types (HR = 2.08, 95% CI: 1.34–3.24), but not in patients with epithelial ovarian cancer (HR = 0.52, 95% CI: 0.08–3.56). Nevertheless, high HHLA2 expression was associated with better OS in patients with pancreatic ductal adenocarcinoma (HR = 0.45, 95% CI: 0.32–0.64). Furthermore, high HHLA2 expression was associated with old age (OR = 1.30, 95% CI: 1.03–1.63), lymph node metastasis (OR = 1.99, 95% CI: 1.41–2.81), and vascular invasion (OR = 1.69, 95% CI: 1.18–2.42).

**Conclusions:**

HHLA2 may serve as a potential prognostic biomarker for solid tumors in Chinese population, by predict the prognosis of cancer patients based on their tumor types.

## 1 Introduction

Malignant tumors have a high incidence and mortality rate, and are the major public health problem worldwide, leading to heavy medical and socioeconomic burdens.^[1]^ Despite substantial advances in surgical, radio- and chemo- therapies in recent decades, poor tumor prognosis remains a tremendous challenge for researchers and clinicians.^[2]^ As a Consequence, the development of novel therapeutics for solid tumors is needed.

Cancer immunotherapy is a rapidly developing cancer treatment designed to stimulate the immune system to fight and eliminate tumors.^[3,4]^ Among various immunotherapy strategies, immune checkpoint blockade seems to be an effective approach.^[5]^ Immune checkpoint proteins are surface molecules on certain immune cell populations that activate or inhibit immune function when engaged to their ligands.^[5]^ Several checkpoint inhibitors have been extensively studied for cancer treatment, and some have been used in clinical trials in cancer patients, such as antibodies against cytotoxic T lymphocyte-associated antigen-4 (CTLA-4), programmed cell death protein 1 (PD-1), or its ligand PD-L1.^[6–8]^ However, the response rate of anti-PD-1 and anti-CTLA4 antibody therapy is rather low in some types of cancer.^[9–12]^ Hence, the development of other immune checkpoint molecules for immunotherapy to improve the survival rate of cancer patients is urgently required.

Human endogenous retrovirus-H long terminal repeat-associating protein 2 (HHLA2), also known as B7H7 or B7-H5, is a newly discovered member of the B7 family.^[13]^ HHLA2 effectively suppresses the activity of CD4+ and CD8+ T cells and reduces the expression of T cell cytokines, such as interferon-γ, tumor necrosis factor-α, interleukin (IL)-5, IL-10, IL-13, IL-17α and IL-22.^[14]^ In addition, HHLA2 is an inducer of the inflammatory response of antigen-presenting cells.^[15]^ HHLA2 is widely expressed in a large proportion of tumor specimens, such as lung cancer, breast cancer, thyroid cancer, ovarian cancer, liver cancer, esophagus cancer, pancreatic cancer, bladder cancer, renal carcinoma, prostate cancer, and colon cancer.^[16]^ Furthermore, recent studies have revealed that high HHLA2 protein expression in primary tumor tissues is associated with poor prognosis of cancer patients.^[13,17–22]^ However, the prognostic significance of HHLA2 is inconclusive.^[9,23–26]^ Thus, we performed this meta-analysis to investigate the association between HHLA2 protein expression and prognosis in patients with cancer.

## 2 Materials and methods

This meta-analysis was performed in accordance with the preferred reporting items for systematic reviews and meta-analyses guidelines.^[27]^ Ethical approval was not required, as the study was conducted based on existing literature and did not involve direct human or animal participation.

### 2.1 Search strategy

A comprehensive electronic search of PubMed, Embase, Web of Science, Chinese National Knowledge Infrastructure, and WanFang databases was performed to identify relevant studies that focused on the prognostic value of HHLA2 in cancer patients prior to November 2020. The following search terms were included “human endogenous retrovirus-H long terminal repeat-associating protein 2” or “HHLA2” or “B7H7” or “B7H5” and “tumor” or “cancer” or “neoplasm” “carcinoma” and “prognosis” or “survival” or “outcome”. The citation lists of the identified studies were also screened for other pertinent studies.

### 2.2 Study selection criteria

Publications were included in this meta-analysis based on the following selection criteria:

1. studies originated from China and focused on Chinese population;
2. tumors were diagnosed by histological or pathological examinations;
3. the expression level of HHLA2 in tumor tissues was detected by immunohistochemistry (IHC) and divided into “positive” and “negative” or “high” and “low” groups;
4. the correlation between HHLA2 expression and overall survival (OS) was assessed;
5. hazard ratios (HRs) with 95% confidence intervals (CIs) were reported or could be calculated based on sufficient data.

Studies were excluded according to the following criteria:

1. reviews, case reports, conference abstracts, letters or editorials;
2. mRNA expression was measured in cancer tissue;
3. patients were not divided into two groups based on HHLA2 expression; and
4. studies without sufficient data to estimate the HR and corresponding 95% CI.

### 2.3 Data extraction and quality assessment

Data were extracted and examined from the included manuscripts by two independent reviews (ZCM and XJ). Any disagreement in the literature assessment was resolved through consensus with a third author (YJ). The following data were extracted: the clinicopathological characteristics, first author's name, publication year, country, cancer type, clinical stage, follow-up time, sample size, cut-off value, number and proportion of patients with high HHLA2 expression, analysis method, language, HR and 95% CI. If univariate and multivariate HRs existed, the latter was selected to minimize bias.

The methodological quality of each recruited study was evaluated independently by two investigators (ZCM and YJ), according to the Newcastle–Ottawa Scale (NOS). The scale included three dimensions as follows: selection (0–4), comparability (0–2), and outcome assessment (0–3), with a total score of 0–9.^[28]^ Articles with a NOS score of ≥ 6 were considered to be of high quality.

### 2.4 Statistical analysis

The Stata version 12.0 software (StataCorp LLC, College Station, Texas, USA) was used to analyze the correlation between HHLA2 expression and OS, and to evaluate the clinicopathological significance of HHLA2 expression in human cancers. Heterogeneity among the included studies was measured by the *I*^2^ and Chi-square Q tests. When significant heterogeneity (*I*^2^ > 50% or *P* < .05) existed, the random effects model was used for the meta-analysis. Otherwise, a fixed effects model was adopted. Subgroup analysis was performed to examine the potential sources of statistical heterogeneity. Sensitivity analysis was conducted by sequentially omitting each individual cohort to evaluate the stability of the results. The potential publication bias was investigated using the funnel plot and Begg/Egger test. A *P-*value of < .05 was considered statistically significant.

## 3 Results

### 3.1 Study selection and characteristics

**Figure 1:**
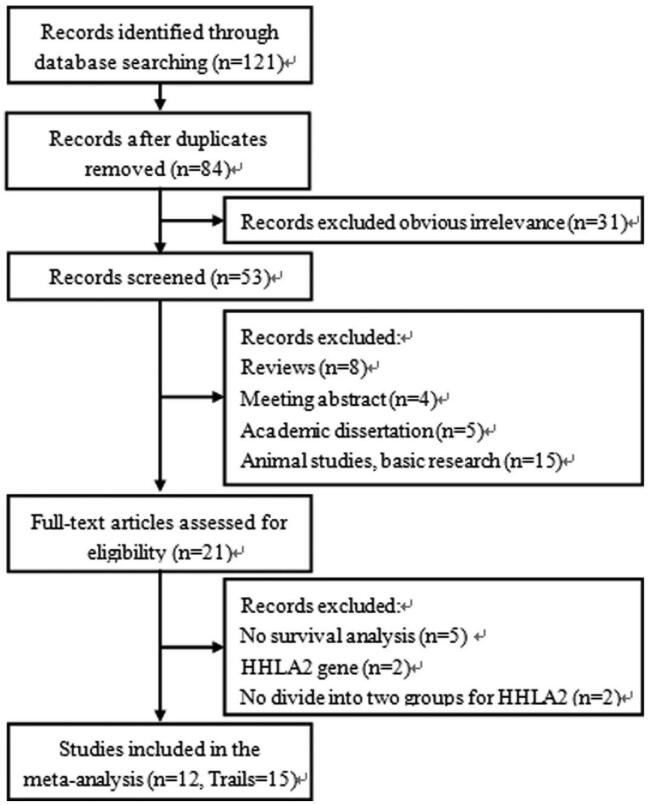
Flow diagram of the study selection process and specific reasons for exclusion of the studies in the meta-analysis.

A flow diagram showing our literature search and screening strategy is presented in Figure 1. A total of 121 articles were initially obtained according to the search strategies. After removing 37 duplicate studies and 31 apparently irrelevant articles, the remaining 53 records were screened by reading the titles and abstracts. Further, 32 studies were excluded (8 for reviews, 4 for meeting abstract, 5 for academic dissertations, 15 for animal studies or basic research). Twenty-one full-text studies were evaluated for eligibility, and finally 12 articles with 15 cohorts were included in the meta-analysis.

All of these studies were published between 2018 and 2020, and all the patients included in these studies were from China. They consisted of the following cancer types: clear cell renal cell carcinoma (ccRCC),^[13,19]^ gastric cancer (GC),^[17,18]^ epithelial ovarian cancer (EOC),^[9,26]^ pancreatic ductal adenocarcinoma (PDAC),^[23,24]^ oral squamous cell carcinoma (OSCC),^[25]^ intrahepatic cholangiocarcinoma (ICC),^[20]^ lung adenocarcinoma,^[21]^ lung squamous cell carcinoma,^[21]^ and colorectal cancer (CRC).^[22]^ The sample sizes ranged from 63 to 403, with a total of 1747 patients. HHLA2 expression was measured by IHC in all cohorts. HRs and the corresponding 95% CIs of OS were obtained by the multivariate analysis in 13 cohorts and univariate analysis or Kaplan–Meier curves in 2 cohorts. The NOS scores of all these studies were between 6 and 8 points, indicating that each article was of high quality. Further detailed descriptions of these eligible articles are listed in Table 1.

**Table 1:** Main characteristics of the eligible studies.

Study	Region	Duration	Cancer type	Clinical stage	Follow up	Number	Detection method	Cut-off value	HHLA2-high (%)	Analysis method	Language	Quality
Zhou QH 2020 (T)	China	2006–2013	ccRCC	I–IV	Until Dec 2019	206	IHC	20%	91 (44.2)	Multivariate	English	8
Zhou QH 2020 (V)	China	2006–2013	ccRCC	I–IV	Until Dec 2019	197	IHC	20%	81 (41.1)	Multivariate	English	8
Wei L 2020	China	NR	GC	I–IV	NR	124	IHC	≥8	66 (53.2)	Multivariate	English	7
Fu Y 2020	China	2009–2013	EOC	I–IV	Until Mar 2018	119	IHC	>31.51%	60 (50.4)	Univariate	English	6
Hu C 2020	China	NR	GC	I–IV	NR	71	IHC	≥8	52 (73.2)	Multivariate	English	7
Chen Q 2019	China	2012–2017	PDAC	I–IV	NR	136	IHC	NR	93 (68.3)	Multivariate	English	7
Yan H 2019	China	2013–2014	PDAC	I–III	Until Nov 2017	92	IHC	>5%	71 (77.2)	Univariate	English	6
Chen L 2019	China	2006–2008	ccRCC	I–IV	NR	87	IHC	>90	26 (29.9)	Multivariate	English	7
Xiao Y 2019	China	2008–2017	OSCC	I–III	NR	201	IHC	85.4	138 (68.7)	Multivariate	English	7
Shi YY 2019	China	2007–2011	EOC	I–IV	NR	64	IHC	>0%	11 (17.2)	Multivariate	Chinese	7
Jing CY 2019 (T)	China	2005–2014	ICC	I–III	Until May 2017	153	IHC	≥5	75 (49.0)	Multivariate	English	8
Jing CY 2019 (V)	China	2005–2014	ICC	I–III	Until May 2017	65	IHC	≥5	44 (67.7)	Multivariate	English	8
Zhang YQ 2018 (LA)	China	2004–2009	LA	I–IV	Until Aug 2017	94	IHC	≥100	31 (33.0)	Multivariate	Chinese	8
Zhang YQ 2018 (LSCC)	China	2004–2009	LSCC	I–IV	Until Aug 2017	75	IHC	≥135	15 (20.0)	Multivariate	Chinese	8
Zhu Z 2018	China	2005–2013	CRC	I–III	Until Aug 2016	63	IHC	>9	30 (47.6)	Multivariate	English	8

### 3.2 Association of HHLA2 expression with OS

**Figure 2:**
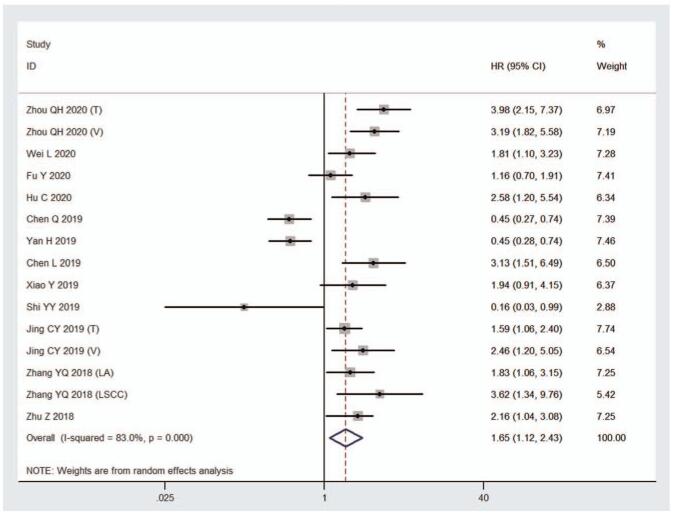
Forest plot of studies evaluating hazard ratios of high HHLA2 expression and the overall survival of cancer patients.

The random effect model was used to calculate the pooled HR and 95% CI because of apparent statistical heterogeneity (*I*^2^ = 83.0%, *P* < .001). The results indicated that high expression of HHLA2 in human tumor tissue was associated with poor OS compared to low expression of HHLA2 (HR = 1.65, 95% CI: 1.12–2.43, *P* = .011) (Fig. 2, Table 2).

**Table 2:** Summary of the meta-analysis results.

Categories	Trials (patients)	HR (95%CI)	*I*^*2*^ (%)	*P*_*h*_	*Z*	*P*_*z*_
OS (All)	15 (1747)	1.65 (1.12–2.43)	83.0	<.001	2.56	.011
Cancer type						
ccRCC	3 (490)	3.42 (2.39–4.91)^F^	0.0	.840	6.70	<.001
GC	2 (195)	2.03 (1.31–3.16)^F^	0.0	.461	3.16	.002
EOC	2 (183)	0.52 (0.08–3.56)	76.4	.040	0.67	.506
PDAC	2 (228)	0.45 (0.32–0.64)^F^	0.0	.960	4.46	<.001
ICC	2 (218)	1.77 (1.24–2.53)^F^	5.4	.304	3.15	.002
LC	2 (169)	2.14 (1.33–3.44)^F^	28.5	.237	3.13	.002
Others	2 (264)	2.08 (1.34–3.24)^F^	0.0	.821	3.26	.001
Clinical stage						
Stage I–IV	10 (1173)	1.76 (1.06–2.92)	83.3	<.001	2.19	.029
Stage I–III	5 (574)	1.46 (0.77–2.77)	84.8	<.001	1.16	.246
Sample size						
≥100	7 (1136)	1.65 (0.97–2.80)	85.2	<.001	1.85	.064
<100	8 (611)	1.64 (0.89–3.02)	83.2	<.001	1.58	.114
HHLA2-high (%)						
≥50%	8 (808)	1.26 (0.77–2.06)	83.7	<.001	0.93	.353
<50%	7 (939)	2.48 (1.65–3.73)	57.3	.029	4.36	<.001
Analysis method						
Multivariate	13 (1536)	1.92 (1.32–2.79)	77.3	<.001	3.42	.001
Univariate	2 (211)	0.72 (0.29–1.81)	85.4	.009	0.69	.489

Considering the significant heterogeneity, we performed subgroup analysis to explore the potential factors that may cause heterogeneity. We classified the included cohorts and conducted subgroup analysis based on cancer type, clinical stage, sample size, proportion of patients with high HHLA2expression, and analysis method (Table 2). Subgroup analysis by cancer type revealed that HHLA2 overexpression was correlated with poor OS in patients with ccRCC (HR = 3.42, 95% CI: 2.39–4.91), GC (HR = 2.03, 95% CI: 1.31–3.16), ICC (HR = 1.77, 95% CI: 1.24–2.53), lung cancer (LC) (HR = 2.14, 95% CI: 1.33–3.44) and other cancer types (HR = 2.08, 95% CI: 1.34–3.24), but not in patients with EOC (HR = 0.52, 95% CI: 0.08–3.56). Nevertheless, high HHLA2 expression was related to better OS in patients with PDAC (HR = 0.45, 95% CI: 0.32–0.64). In addition, subgroup analysis according to cancer type showed that there was no significant heterogeneity within each subgroup, except for the EOC subgroup. When subgroup analysis was performed according to clinical stage, sample size, proportion of patients with high HHLA2 expression and analysis method, the heterogeneity within each subgroup did not change significantly. Therefore, cancer type may be the source of heterogeneity.

### 3.3 Sensitivity analysis and publication bias

**Figure 3:**
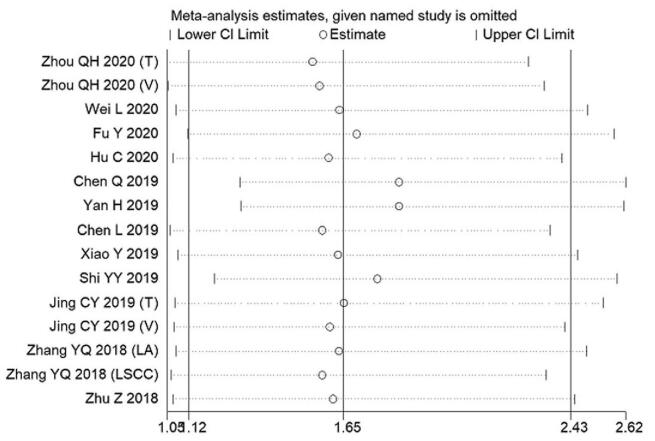
Sensitivity analysis for evaluating the effects of individual studies on the relationship between HHLA2 expression and overall survival in patients with solid cancer.

**Figure 4:**
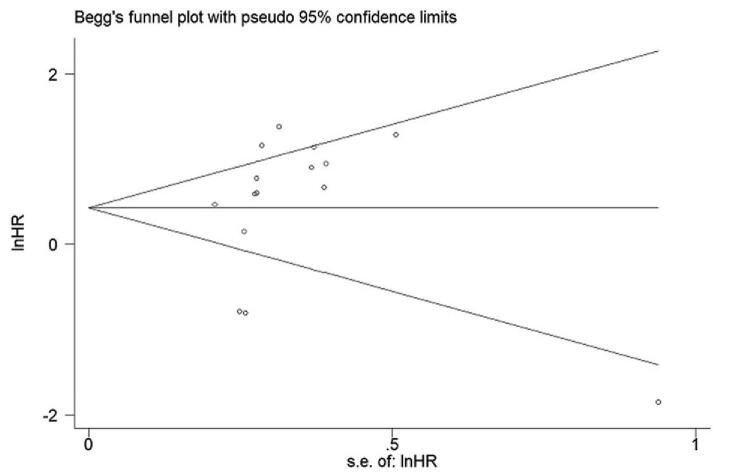
Begg's funnel plots for assessing potential publication bias on the relationship between HHLA2 expression and overall survival in patients with solid tumors.

We performed sensitivity analysis by successively ignoring individual cohort studies, and the results showed that no individual cohort affected the relationship between HHLA2 expression and OS in patients with solid tumors (Fig. 3). Moreover, no publication bias was detected, which was verified by the Begg test (*P* = .075), Egger's test (*P* = .507), and funnel plot (Fig. 4). Therefore, our results were robust and reliable.

### 3.4 Association of expression with clinicopathological parameters

Meta-analysis of the association between HHLA2 expression and clinicopathological parameters showed a significant relationship between high HHLA2 expression and old age (OR = 1.30, 95% CI: 1.03–1.63), lymph node metastasis (OR = 1.99, 95% CI: 1.41–2.81), and vascular invasion (OR = 1.69, 95% CI: 1.18–2.42). However, a similar relationship was not observed between HHLA2 expression and gender (OR = 1.21, 95% CI: 0.91–1.61), tumor size (OR = 1.28, 95% CI: 0.86–1.90), clinical stage (OR = 1.68, 95% CI: 0.94–3.00), tumor depth (OR = 1.67, 95% CI: 0.78–3.59) and distant metastasis (OR = 1.52, 95% CI: 0.83–2.79). (Table 3)

**Table 3:** Meta-analysis of HHLA2 and clinicopathological features in cancer patients.

Categories	Trials (Patients)	OR (95%CI)	*I*^*2*^(%)	*P*_*h*_	Z	*P*_*z*_
Age (young vs old)	12 (1308)	1.30 (1.03–1.63)	0.0	.525	2.24	.025
Gender (male vs female)	9 (1004)	1.21 (0.91–1.61)	0.0	.780	1.34	.181
Tumor size (small vs large)	5 (465)	1.28 (0.86–1.90)	38.4	.165	1.20	.231
Clinical stage (I–II vs. III–IV)	13 (1391)	1.68 (0.94–3.00)^R^	77.7	<.001	1.77	.077
Tumor depth (T0–T2 vs T3–T4)	6 (510)	1.67 (0.78–3.59)^R^	58.6	.034	1.32	.188
Lymph node metastasis (negative vs positive)	9 (787)	1.99 (1.41–2.81)	26.5	.208	3.93	<.001
Distant metastasis (negative vs positive)	7 (611)	1.52 (0.83–2.79)	24.9	.239	1.36	.174
vascular invasion (negative vs positive)	7 (901)	1.69 (1.18–2.42)	0.0	.548	2.86	.004

## 4 Discussion

In the past several decades, many studies have focused on identifying novel diagnostic and prognostic markers of tumors in order to advance treatment and outcomes by providing information for clinical decision-making.^[29–33]^ The prognostic significance of HHLA2 expression has been evaluated in a variety of solid tumors. Here, we aimed to summarize and evaluate the results of published studies and extract valuable information that can be used in clinical decision-making for human solid tumors.

A total of 12 studies, including 15 cohorts and 1747 patients, were included. Our results demonstrated that high HHLA2 expression predicted poor OS in Chinese patients with cancers. Furthermore, sensitivity analysis and publication bias proved that the results were robust and reliable. However, a significant heterogeneity existed among these studies. Considering the apparent heterogeneity, subgroup analysis was performed. Subgroup analysis showed that clinical stage, sample size, proportion of patients with high HHLA2 expression and analysis method did not change heterogeneity significantly. Subgroup analysis by cancer type showed that HHLA2 overexpression was correlated with poor OS in patients with ccRCC, GC, ICC, LC, and other cancer types, but not in patients with EOC. Nevertheless, high HHLA2 expression was related to better OS in patients with PDAC. In addition, the cancer type subgroup significantly reduced the heterogeneity within each subgroup. Thus, cancer type was the main source of heterogeneity and HHLA2 expression may play a different role in different types of cancer, especially in patients with PDAC. The mechanism should be further studied to reveal the influence of HHLA2 expression on the types of cancer.

HHLA2 is involved in tumor immunosuppressive mechanisms and is a checkpoint for inhibition.^[9]^ HHLA2 inhibits CD4 + and CD8 + T cell proliferation and cytokine production by binding to putative receptors in various immune cells.^[34]^ In addition, HHLA2 binds to receptors on T cells and other immune cells to evade immune attacks.^[35]^ Furthermore, HHLA2 binds to CD28H, which is expressed in naive T cells, natural killer cells, endothelial cells and epithelial cells, but not in activated T cells, and plays an important a role in angiogenesis, cell-cell interaction, and cell migration.^[36,37]^ However, the HHLA2/CD28H interaction can also co-stimulate T cell proliferation and cytokine production via the AKT pathway.^[18,38]^ Therefore, HHLA2 may have dual immune functions depending on the immune environment, receptor engagement or blockade, or interaction with different receptors, which is similar to the other members of the B7 family, such as B7-H3 and B7x.^[24,39]^ In view of the conflicting results shown in HHLA2 so far, continued research and further understanding of these pathways is urgently needed to understand the causes of these differences among different tumors, to provide new strategies for tumor immunotherapy.

Several limitations should be noted in the present study. First, all the patients included in this study were from China, which affected the applicability of the results to a certain extent. Second, the cut-off values of high HHLA2 expression were inconsistent, which may affect the effectiveness of HHLA2 as a predictor of cancer prognosis. Third, HR and 95% CI in some studies were calculated by extracting data from Kaplan–Meier curves rather than directly from the original literature, which inevitably led to small statistical deviations. Fourth, HHLA2 may act differently in patients with PDAC than in other cancer types, and PDAC patients were also included in the study. Given the limitations of this study, further well-designed studies that include evaluation of more tumor types with a larger sample size are needed.

In summary, high HHLA2 expression was associated with poor OS in Chinese patients with solid tumors, and might be used as a potential prognostic marker and tumor treatment target. However, high HHLA2 expression was related to better OS in patients with PDAC. Therefore, more mechanistic studies are needed for further analysis.

## Author contributions

**Conceptualization:** Xiaohong Zhang.

**Data curation:** Chuanmeng Zhang, Jie Xu.

**Investigation:** Chuanmeng Zhang, Jie Xu, Jun Ye.

**Software:** Chuanmeng Zhang, Jie Xu.

**Supervision:** Xiaohong Zhang.

**Writing – original draft:** Chuanmeng Zhang, Jun Ye.

**Writing – review & editing:** Chuanmeng Zhang, Xiaohong Zhang.
